# NEWS – RESOURCES

**DOI:** 10.7189/jogh-08-010203

**Published:** 2018-06

## Demography

➢ Figures from China’s national statistics bureau show that 17.23 million births were recorded in 2017, down from 17.86 million in 2016. This is the first fall since China relaxed its one-child policy – which allows all couples to have 2 children – and indicates that the reform has not halted falling fertility rates which could endanger its long-term development. The spike in births (an increase of 1.3 million) in 2016 could be a one-off, as couples who had been waiting to have a second child did so when the policy changed. By the mid-21^st^ century, 1-in-3 Chinese people is forecast to be aged more than 60 – the rapid ageing of the population is creating a shortage of workers and places a burden on social services whilst per capita incomes lag behind developed nations. Some analysts believe that shifting attitudes, such as more emphasis on children's education, are lowering China's birth rate, which is also depressed by the rising average age at marriage. There are calls for tax breaks and subsidies to encourage couples to have more children. China's 2-child policy has affected the country's urban residents more than its rural residents, as the 1-child policy was less stringently enforced in the countryside; however urban residents tend to have fewer children. This is partly because they perceive the costs of raising a child to be prohibitive, and because women in cities tend to be more educated. (Financial Times, 18 January 2018)

➢ According to Statistics Korea, South Korea’s birthrate has fallen to a record low, with 357 700 babies born in 2017 – an 11% decrease from the previous year, and the first time the number of yearly births has fallen below 400 000. South Korea’s fertility rate has fallen to 1.05 children per woman. It has taken South Korea 15 years to reach fertility rates that other countries have taken 100 years to achieve. Experts identify multiple reasons behind the country’s falling birthrate, including falling numbers of people aged 20-39 years, high costs of bringing up a child, high youth unemployment. There are potential problems associated with South Korea’s low birth rate and ageing population, including rising pension costs, economic stagnation, and tax increases to fund services. South Korea’s government has spent US$ 89 billion to increase the country’s birth-rate, but this has largely failed. One reason behind this failure is that couples were encouraged to have 3-4 children, where it might have been more effective to encourage couples to have their first children. (KBS World Radio, 5 March 2018)

➢ Globally, the average person’s lifespan has risen almost everywhere, with China, the USA and most of eastern Europe seeing average life expectancy in the high 70s, and people in Western Europe and Japan can expect to live into their early 80s. However, according to a survey from UBS Financial Services, the wealthiest individuals (mainly those with investable assets of at least US$ 1 million) are expecting to live to 100 years. This may not be implausible, as the average Japanese woman now has a life expectancy of 87, and wealthier people often have a built-in longevity advantage. Eg, the richest 1% of women in the USA live for 10 years longer than the poorest 1%, and the gap between the richest and poorest American men is 15 years. Moreover, it appears that these individuals are making financial adjustments to take account of increased life expectancy, and indicate willingness to spend part of their fortunes on extra years of life. Mortality rates in higher-income groups in the US are much lower than other US groups, showing that inequality is more than widening gaps between health and income – the richest people in the US may be living much longer, healthier lives than their fellow US citizens. (Bloomberg, 20 April 2018)

➢ Nearly 10% of global gross domestic product is spent on health care, with rich countries spending an average of 12%, middle-income countries 6%, and low-income countries just under 6%. In developed countries, 60% of health spending is from public sources, but in poorer countries, this falls to 40%. However, the variation in health spending patterns across less developed countries means that people have to cover gaps in health costs by themselves (also known as user fees or out-of-pocket expenses). These fees can prevent people who most need care from seeking it, and out of the US$ 500 billion generated each year by user fees, the World Bank estimates that 40% is wasted. More than 110 countries have some form of social health-insurance, supplemented by voluntary private insurance and user fees. However, this falls short of universal health coverage, as healthy people avoid voluntary schemes. Countries that seek to expand coverage towards universal health care can take two different approaches. The first approach is to offer in-depth coverage to small groups, and then work outwards. However, these leaves groups without insurance, and those with coverage have no incentive to help them get it. The second, better approach, is to cover more people, but begin with more limited benefits. Eg, 90% of Rwanda’s citizens have health insurance, plus various treatments partly covered by the Global Fund. This has cut user fees and improved health outcomes – between 2000 and 2011, the TB mortality rate fell from 50 to 14 per 100 000 people. Poorer countries could also expand their tax base, eg, extractive industries and tobacco, alcohol and air pollution. Cost-effective aid can fight communicable diseases whilst building health systems and expanding coverage. As aid from Western countries falters, poorer countries will need to step up their own efforts towards universal health coverage, and to ensure that the SDG’s health-related targets will be met on time. (Economist, 26 April 2018)

➢ At a forum organisation by the Development Communications Network, in collaboration with the Nigerian Urban Reproductive Health Initiative in Ibadan, Dr Muideen Olantunji said that Nigeria likely to become the 3rd-largest country in terms of population by 2050. Its population could reach 450 million, behind India and China. In 1999, Nigeria’s population was roughly equivalent to Italy, France or the UK. Dr Olantunji noted that Nigeria aims to increase its Contraceptives Prevalence Rate (CPR) to 36% (or 11.6 million women), and that mid- and long-term methods to reach this goal will prevent unwanted pregnancies, and reduce maternal and infant deaths. This would result in over 7.4 million unwanted pregnancies avoided, and over 650 000 maternal and infant deaths avoided. (New Telegraph, 4 May 2018)

## Economy

➢ African millennials have been heavily drawn to cryptocurrencies such as bitcoin, and part of their appeal lies in their lack of upfront costs and low time investments - important when many such millennials have side-lines to compensate for limited earning opportunities and high unemployment. In some cities across Africa, people are turning to cryptocurrencies as a cheaper way of transferring funds across borders. Established money transfer providers, such as Western Union, transfer the local currency into US$, which then have to be converted back to the local currency by the recipient. Cryptocurrencies can substitute for the US$, reducing the transaction costs (especially when there is a US$ shortage, or restrictions on accessing US$). In Nigeria, when the government restricted access to US$ during a financial crisis, businesses could transfer cash abroad using cryptocurrencies. Countries such as Zimbabwe, with political and economic instability, use cryptocurrencies to store value, buy goods and services from abroad, and for overseas remittances. However, many central bankers have warned against the instability of cryptocurrencies, warning to “use what you can afford to lose.” Some digital security experts see the main innovation in cryptocurrencies – blockchain – as their most important feature, with potential to tackle the shortage of banking facilities, corrupt voting systems and secure digital identities, amongst others. (BBC, 8 January 2018)

➢ India’s population is set to overtake China’s, and occasionally its economic growth matches that of China. Many companies expect India's middle-class to swell to 300-400 million people, with the resultant potential for increased consumption. However, India's middle-class is a minuscule part of its overall population; the top 1% of adults earn at least US$ 20 000 a year, making them equivalent to Hong Kong in terms of population and average income. The next 9% is akin to central Europe, the next 40% reflects the combined wealth of Bangladesh and Pakistan. The remaining 50% of India's population are akin to the poorest regions of Africa. India's chances of growing its middle-class is being throttled by rising inequality, where the top 1% of earners took nearly 33% of all additional income generated between 1980 and 2014. The richest Indians are 10 times richer now than in 1980, whilst the income of median earners has risen much more slowly, not even doubling in that period. Although India has done well in moving people from earning US$2/d to US3/d, middle-earners in countries at India's stage of economic development usually benefit more from economic growth. 93% of Indians work in unproductive small or micro-enterprises, making it hugely difficult for India to follow China's lead in creating middle-class jobs from factory output. Companies need to focus more on how to help India's consumer to gain access to new goods, eg, cheap mobile data can drive its streaming services, and the country will have to work hard to turn potential into profits. (Economist, 11 January 2018)

➢ There are 222 million people aged over 60 years in China, and if they created their own country, it would be the 5th largest on earth; and its population would increase to 300 million by 2025. However, China has low levels of pension fund assets relative to its economy, with just US$ 177 billion of pension assets in 2017 – or 1.5% of its GDP. This figure may well be an underestimate due to scarce data, but pension provision is still low by international standards. If China shifted its high savings rate into funded pension infrastructure, the global demand for stocks and bonds - assets commonly bought by pension funds – would increase markedly. There are signs that this is already happening, with China experiencing the fastest compound annual growth rate of pension assets over the past 5 years, and assets managers are beginning to pay attention to China’s mutual and pension fund business. Increasing pension assets in China would likely raise demand for securities, and offshore investing would rise. The international impact depends on the future liberalisation of China’s markets, and the ease of money flows, both on- and off-shore. China’s growing middle-class seem likely to demand a more developed pension system, and the one-child policy means that a couple could potentially have four elderly parents to support, further increasing demand for pension reform. (Financial Times, 14 February 2018)

➢ Mr Gregory Rockson, a Ghanaian entrepreneur, has developed and launched mPharma, an electronic prescription system that aims to overcome some of the problems besetting Africa’s pharmaceutical chain, including sprawling supply chains, low order volumes and high prices. mPharma is a “pharmacy benefits manager” that buys medicines on behalf of pharmacies, improving certainty for suppliers and pharmacists, and, by providing working capital and the increasing the volume of purchases, it could negotiate lower prices with suppliers. mPharma generates revenues from a commission on the drugs it purchases, and has also launched Mutti, an electronic credit financing service to help patients pay for health care. Mr Rockson aims to make mPharma profitable within 3 years, and sees potential to generate income from sales of its data on medicines use. Mr Rockson has raised US $10 million in investment, which he plans to fund expansion over the next 2 years. mPharma spans 110 pharmacies in Ghana, Nigeria, Zambia and Zimbabwe, and has served an estimated 100 000 patients to date. “Our goal is to build the largest African drug retail support network outside South Africa in the next five years … the question for me is now not about how we make money but how do we build enough scale, fast enough,” said Mr Rockson. (Financial Times, 12 March 2018)

➢ Following Beijing’s threat to impose tariffs on imports, US soybean sales to China ground to a halt, amidst other signs of growing trade tensions upsetting the global flow of commodities. Soybeans are the US’s most valuable agricultural export to China – worth US $12 billion in 2017, but at a current price of US$ 420 per tonne, a potential tariff would translate as a tax of US$ 100 per tonne on shipments, with no way of managing the risk of increased prices and price instability. Trade figures shown that US soybean sales to China fell by 10% over the past 4 weeks, from the same period in 2017. Worldwide, growing trade disputes are disrupting agricultural supply chains, causing US farmers and manufacturers to step back from expansion plans due to steel and aluminum tariffs. In a separate move, China has imposed anti-dumping deposits on US imports of sorghum, used for livestock feed. (Strait Times, 3 May 2018)

## Energy

➢ Access to clean and affordable energy underpins many of the UN’s Sustainable Development Goals, but more than 40% of people in low-income countries are without access to electricity. Accelerated clean energy access would provide economic, social and environmental benefits, but investment in renewable energy and energy efficiency must increase 5-fold if universal energy access is to be achieved by 2030. Amongst least developed countries, Senegal has made significant progress in expanding energy access, with nationwide access above 50%, and 85% in cities. To achieve universal access, the UN states that Senegal must double its electrification rate, but it is expensive to connect under-served rural areas to Senegal’s centralised grid. To tackle these problems, Senegal is tapping into its potential for solar energy, and setting up micro-grids (generators which can be run independently or connected to the national grid). In 2017, Senegal opened Africa’s largest solar power facility, which is capable of supplying 215 000 citizens each year – with other solar projects in the pipeline. The World Bank is also supporting Senegal’s government with relatively large micro-grids that cover larger areas, alongside helping entrepreneurs to build smaller micro-grids in rural areas. (The Conversation, 14 January 2018)

➢ Coal supplied 80% of India's total power source in 2016-17, but for the first time, new renewable energy sources are cheaper to build than running most existing coal-powered plants, and wind and solar energy are 20% cheaper that the wholesale price of coal. The cost of renewable energy in India has fallen by 50% over 2 years, and this trajectory is expected to continue. 2016-17 may have been the tipping-point for this trend, when the capacity of renewable energy sources doubled. India's demand for power is set to double over the next 10 years, and its draft National Electricity Plan calls for the increased demand to be met with 275 gigawatts of renewable energy capacity, without requiring new coal plants beyond those already under construction. India's coal plants already run at 50% capacity, and nearly all violate air pollution standards. Moreover, India's Central Electricity Authority has proposed closing nearly 50 gigawatts of coal capacity by 2027. Ensuring that the remaining coal plants comply with environmental legislation will be expensive, so operating already uneconomic plants will get more expensive as they run less frequently and generate less profit. (Forbes, 30 January 2018)

➢ The Russian energy giant, Gazprom, announced that gas from its Siberian fields could start moving through pipelines to China over the next 5 years, showcasing Russia’s efforts to develop its gas sector in as many geographical locations as possible. Gazprom already has a 30-year sales agreement with the China National Petroleum Corporation (CNPC), that plans for 1.3 trillion feet^3^ (28.32 L^3^) of natural gas through the Power of Siberia gas pipeline. CNPC is the 3^rd^ largest oil company in the world, and China’s largest oil and gas producer and supplier. Gazprom confirmed that the Power of Siberia pipeline was 75% completed, and gas shipments to China are scheduled to begin in December 2019. Gazprom’s announcement coincides with increasing demand for natural gas in China, amid a government mandate that at least 10% of China’s energy mixed used for power generation to be derived from gas, as China tackles air pollution by replacing coal-fired power stations with gas, as well as for industrial and manufacturing end-users. China’s liquid natural gas imports reached record levels in January 2018, and worldwide it is the fastest growing market for the product. (Oilprice.com, 22 March 2018)

➢ The World Health Organization has released data that shows more than 90% of the world’s population is breathing in high levels of pollutants, which causes 7 million deaths each year. Although air pollution is a global problem, the poorest and most marginalised people are worst-affected, with most of the 7 million deaths occurring in low- and middle-income countries, especially in Asia and Africa. The use of polluting cooking fuels (eg, charcoal) indoors, is a major source of pollution, and causes an estimated 3.8 million premature deaths each year – and more than 40% of the global population are still without access to clean cooking fuels and technologies. Although access to clean cooking fuels is improving, it is not keeping pace with population growth. Outdoor air pollution is linked to 4.2 million deaths each year, and around a million deaths are a combination of indoor and outdoor pollution. (NDTV, 2 May 2018)

➢ *Reuters* has reported that the threat of US oil sanctions against Iran has led to the Russian oil giant Lukoil to put planned investments in Iran on hold, whilst the Indian company which owns the world’s largest oil-refining complex plans to stop buying oil from Iran. The US announced in May that it will reinstate sanctions against Iran, which had earlier been lifted in exchange for curbs on Iran’s nuclear activities. The French energy giant Total has also announced that it will abandon a major gas project in Iran unless it gets a US sanctions waiver. Russia and France, both signatories to the nuclear deal with Iran, have pledged to uphold it and are working with Tehran to find avenues to enable their businesses to by-pass US sanctions. However, these companies face the possibilities of fines worth US$ billions if these avenues are not water-tight. India, which was not a signatory to the nuclear deal, has announced that it will not honour US sanctions, although some companies are reputedly wary of US sanctions and will independently stop oil imports from Tehran. Global insurers are voicing concerns about the risks of doing business with Iran, and some global shipping lines are not taking new orders for oil shipments from Iran. Iran’s government has said that it will honour the nuclear deal provided it can sell its oil freely around the world. Iran’s crude oil exports fell by 100 000 barrels/d in May – from its record high of 2.6 million barrels/d – in signs that the threat of US sanctions might be deterring buyers. (Radio Free Europe, 31 May 2018)

## Environment

➢ UNICEF has called for urgent government action to limit air pollution in Mongolia's capital, Ulaanbaatar, claiming that smog is causing a public health crisis, especially amongst children and pregnant women. Smog heightens the risk of stillbirth, premature birth, lower birth weight, pneumonia, bronchitis, asthma, inhibited brain development and death. According to UNICEF, Ulaanbaatar's air pollution is worse than cities such as Beijing and New Delhi, with average concentrations of breathable airborne particles (known as PM2.5) as high as 3320 µg per m^3^ at one station - the WHO recommends annual average PM2.5 concentrations of no more than 10 µg, and Beijing's PM2.5 levels were 34 µg at the same time. Ulaanbaatar's population has doubled over the past 20 years, due to herders migrating from the countryside, and many such households burn coal or rubbish for warmth, leading to smog levels to spike in the winter months. (Channel News Asia, 23 February 2018)

➢ Motor vehicles are a major source of pollution in China’s cities, and air pollution causes up to one million excess deaths each year in China, according to statistics from the World Health Organization. In order to achieve improved air quality by 2035, systematic change is needed – and this is coming from China’s auto industry. In 2017, China produced 680 000 all-electric vehicles – more than the rest of the world combined – and its growth in output of these vehicles is outstripping the rest of the world, too. These vehicles are battery electric vehicles (BEVs), which are not hybrid vehicles with a back-up petrol engine. To meet the challenges of vehicular power range, China is pioneering the mass development of public charging piles, with 214 000 in place at the end of 2017, and plans to have 500 000 charging piles in place by 2020. The logistics of these charging piles differ from petrol stations, as electrical charging can take up to 30 minutes, so an electric car ecosystem needs widely distributed charging piles, to charge parked cars. (Forbes, 6 March 2018)

➢ In 2017, Britain’s CO_2_ emissions from fossil fuels fell by 2.6% - mainly due to 19% decline in coal usage from coal-fired plants, bringing the country’s carbon emissions to levels last seen in the 1890s. Britain’s CO_2_ levels are currently 38% below 1990 levels, and have been declining steadily since 2012, with big falls in 2014 and 2016. However, in order to meet its climate targets over the next few decades, Britain must maintain and even accelerate this rate of decline, focusing on the constructive and transport sectors, where emission reductions are elusive. Indeed, the motor industry saw the average emissions for new cars rising for the first time since 2000. This could be caused by a manufacturing trend towards SUVs, and a lack of tax incentives for cleaner electric and hydrogen cars. Previously, low-emission vehicles were zero-rated for tax purposes, but in 2015 all vehicles (including hybrid vehicles, but excluding electric and hydrogen cars), were placed in the same tax bracket. This meant that a hybrid Toyota Prius was taxed at the same rate as a Porsche, both reducing incentives for drivers to buy cleaner cars, and pressure on manufacturers to supply them. (BBC, 7 March 2018)

➢ Over 100 people were injured and 72 people have died as dust storms and heavy rains have swept across northern India, in the states of Uttar Pradesh and Rajasthan. Winds as high as 126 km/h were reported during the dust storms, which were following by heavy rainfall, as much as 48.2 mm within 3 hours. Hundreds of animals were also killed, trees were uprooted, and building and infrastructure were destroyed. Dust storms are common occurrences in Rajasthan prior to monsoon rains, as the state contains desert – and dry, sandy soils are prone to wind erosion and transportation. However, many parts of northern Pakistan and India have reported extremely high temperatures – Pakistan’s Sindh province recently recorded the world’s highest temperature of 50.2°C – so it is possible that the temperatures induced higher wind circulation, and a trough of low pressure may have introduced moisture into the atmosphere, encouraging thunderstorms to develop. (Al Jazeera, 3 May 2018)

➢ As the death toll from drinking contaminated water in Cambodia’s Kratie district rose to 18, scores of indigenous ethnic Phnorng residents of four villages in east Cambodia’s Mondulkiri province have fallen ill from drinking contaminated water. More than 50% of the people affected in Mondulkiri have received hospital treatment after paying fees of US$ 50-100, whilst the others, unable to cover the fees, are resting at home and using traditional remedies. The water is from a stream allegedly contaminated by a Chinese mining company drilling upstream near the water source. According to the US-based advocacy group, Water.org, about 4 million Cambodian people lack access to clean water, and 6 million people have inadequate access to sanitation and hygiene. The Sre Chhouk commune chief, Te Khit, denied that the illnesses were caused by contaminated water, stating that investigations by provincial authorities and officials from Cambodia’s Ministry of Health found that the illnesses were influenza, and that residents have been educated about proper water sanitation and have received clean drinking supplies. (*Radio Free Asiaa*, 15 May 2018)

## Food, Water and Sanitation

➢ Over the past two years, Somalia has experienced a regional drought which has displaced more than 1.4 million people, many of them farmers and pastoralists, who have sought refuge in cities such as Baidoa in southwestern Somalia. To support these people, aid agencies have relied on cash transfers, which can include direct transfers to mobile phones through mobile money, vouchers, or bank-cards. Cash transfers were scaled-up in Somalia in 2017, with 3 million people – around 25% of its population – receiving some form of cash assistance. The World Food Programme has increased its proportion of cash-based food aid from 10% in 2015 to 60% in 2017. Amidst the massive displacement, aid agencies began to improve their cash transfers distribution, to avoid duplication of effort. Some agencies have begun to envisage the current cash transfers evolving into a national social safety net to assist people beyond the current food crisis, allowing relief efforts to be quickly scaled-up during the next emergency. Cash transfers allow people to buy what they most need, and are more efficient than food distribution – in Somalia, about 80% of cash transfers reach the beneficiary, compared to 60% of in-kind food aid. Cash transfers work well in Somalia, because its private sector is still functioning, with reasonably-stable markets for foods thanks to food imports; other countries, such as Yemen and South Sudan, have non-functioning markets and cash transfers work less well as a result. (Devex, 26 January 2018)

➢ Malawi has experienced 434 recorded cases of cholera since the outbreak in November 2017, with six people dying. Cholera was first reported in the border area of Karonga, where four people died. UNICEF is concerned over Malawi’s rising number of cholera infections, calling for improved hygiene and sanitary measures as a matter of urgency. In Lilongwe, some people are using contaminated water from shallow wells and bore holes, rather than from the Lilongwe Water Board. Malawi’s Health Minister, Atupele Muluzi confirmed that the country will receive 216 000 oral vaccines from the World Health Organization, which will be distributed in to 108 000 people in Karonga. Another 450 000 doses are expected to arrive later, and will be distributed to other cholera-affected regions in Malawi. (Xinhua, 12 February 2018)

➢ A 10-year study of Chinese agriculture has analysed vast amounts of data to develop improved agricultural practices. The improved practices were forwarded to farmers, with 20.9 million farmers adopting the recommendations. China’s farmers use 305 kg of nitrogen per hectare/y – more than 4 times the global average, and the project aimed to reduce nitrogen usage without decreasing yields. The researchers conducted field studies, and used the data to develop evidence-based advice grounded in local conditions. Across the farmers who adopted study recommendations, crop production increased by an average of 11%, whilst fertiliser use fell by 15% per crop, saving 1.2 million tonnes of nitrogen. Whilst lessons from the project could be applied to other countries, and help improve agricultural productivity to support the world’s growing population, some researchers point to some lessons being difficult to transfer, as many farmers in other low- and middle-income countries have inadequate supplies of fertiliser, so are not affected by the same problems of nitrogen overuse. (Nature, 7 March 2018)

➢ Diarrhoea kills up to 500 000 children each year, and repeated infections may also weaken children, and stunt their development. However, Bangladesh is making huge strides against diarrhoea and other enteric diseases, with some areas showing a 90% reduction in deaths over the past 20 years. This, combined with far-reaching immunisation programmes and steady economic growth, has reduced Bangladesh’s mortality rate for children aged under 5 years to 16% below the global average – in 1990, it was 54% higher. Part of Bangladesh’s success is due to improved sanitation. Bangladeshi villages are studded with small pit latrines and tubewells for water – showing that simple, imperfect solutions can reap dividends. India, roughly twice as wealthy as Bangladesh, has subsidised and built many latrines, but open defecation remains widespread. Bangladesh has been successful in changing people’s behaviour – from mothers washing their hands before feeding their children, and the realisation that installing latrines in poorer people’s homes encouraged richer people to adopt their use, and not *vice versa.* (Economist, 22 March 2018)

➢ The recent water crisis in Cape Town, South Africa, threatened to make South Africa’s second city, with its population of nearly 4 million people, almost completely dry. Globally, Cape Town is the first major city to face such a scenario. However, “Day Zero”, the anticipated moment when city engineers would turn off the city’s taps and forcing the population to queue at military-guarded standpipes, with water rationed to 25 L per person, has been pushed back from April 2018 into 2019. The arrival of Day Zero was predicated on falling annual rainfall - from 1100 mm in 2013 in 500 mm in 2017, devastating the province’s supply system, which is almost entirely reliant on surface water. Day Zero was averted by drastic civic water conservation measures, with residents halving their water consumption, and sectors such as agriculture and tourism bearing the brunt of water shortages. Day Zero would have been triggered when dam levels fell to 13.5% of capacity - they are currently at 19%. City authorities used a mixture of nudge tactics (eg, showing colour-coded maps of water usage, and increasing tariffs on wealthier people to pay for water supplies for poorer people), with the overall aim of persuading a divided society to think of water shortages as a common problem. Some people in shanty townships pointed out that water restrictions would not apply to them – their water consumption was already at or below the 25 L ration. In 1996, South Africa’s new democratic constitution included a right to water for all. “What Cape Town’s crisis has made clear is that we are very far from realising that right,” said Melita Steele, a Greenpeace campaigner. (Financial Time*s*, 1 May 2018)

## Peace and Human Rights

➢ Ethiopia’s prime minister, Mr Hailemariam Desalegn, has announced the release of political prisoners, and the closure of Maekelawi, a notorious detention centre. Human rights groups have previously accused the government of using anti-terrorism laws to jail its critics, and those imprisoned including opposition activists at the centre of anti-government protests in 2015 and 2016, and journalists who have criticised the government. Although it is difficult to know how many political prisoners are held in Ethiopia, it is estimated that 1000 people are held under its anti-terrorism laws, with another 5000 trials pending following the declaration of a state of emergency in October 2016. The government has not yet confirmed which prisoners will be released, and who is deemed a political prisoner, or a terrorist. The detention centre, Maekelawi, was allegedly used for torturing prisoners – this is strongly denied by the Ethiopian government – and will be replaced with a modern detention centre, which will meet international standards, according to Mr Desalegn. Amnesty International welcomed Mr Desalegn’s announcements, although stated that the closure of Maekelawi should not be used to “whitewash” the “horrifying” events that took place there. (BBC, 3 January 2018)

➢ A hearing in South Africa was told that 144 mentally-ill patients died, during and after the Gauteng health department’s decision to move them from the Life Esidimeni care facilities to the facilities of various other non-governmental organisations. The investigation also heard the testimony from Prof. Christoffel Grobler, a psychiatrist, who confirmed that patients – contrary to legal requirements – were not consulted before the moves, and that there were risks in moving these patients to the NGO facilities. He also stated that patients’ families should be informed before any transfers or other major procedures are undertaken, but these consultations likewise also did not take place. Qedani Mahlangu of Gauteng’s health department, defended her department’s actions, saying that she was not a “prophet”, and could not have anticipated the deaths of the patients. “It was not intentional. If I had foresight like a prophet, maybe I would have seen, but I am not a prophet. I do not even know what will happen tomorrow.” Prof. Grobler, in a counter-statement said, “one does not need to be an expert nor a prophet to know that there was going to be drama with the moving of patients.” (news24.com, 26 January 2018)

➢ Turkey’s president, Mr Tayyip Erdogan, announced that the government will remove the word “Turkish” from the country’s main medical organisation, the Turkish Medical Association (TMA). This follows the TMA’s criticism of Turkey’s offensive in northwest Syria’s Afrin district. Mr Erdogan accused the TMA of treason, and 11 of its senior members were detained in January (although released on probation). According to Mr Erdogan, “this institution has nothing to do with Turkishness and nothing about them is worthy of the notion of Turkishness.” The TMA, which represents 80% of Turkey’s doctors, said that the right to call itself “Turkish” was enshrined in its constitution, and reflected its service to the public good. The government may also be moving towards TMA membership no longer being a requirement to practice medicine in a private practice, alongside bar membership no longer being required to practice law – both of which could weaken two influential groups that have frequently opposed Mr Erdogan. The main opposition party, the Republican People’s Party, said these moves were designed to further polarise the country, and called for them to be abandoned. Since a failed coup in 2016, 50 000 people have been detained, and 150 000 suspended or sacked from their jobs. (Reuters, 8 February 2018)

➢ 22 February 2018 is the 75th anniversary of the execution of students Christoph Probst and siblings Hans and Sophie Scholl, members of the White Rose group. The three young people were arrested and executed in 1943, for their role in writing and distributing the White Rose’s anti-Nazi leaflets in war-time Germany. Their executions were followed by the arrests and executions of other members of the group later in 1943. The White Rose sent thousands of copies of each of their leaflets denouncing the Nazi regime and its crimes across Germany. It was a rare act of resistance in Nazi Germany, and their prescient leaflets condemned Hitler and warned that Germany would be outcast for the crimes being committed in its name. Another leaflet blamed the Nazi regime for the deaths of 330 000 German men at Stalingrad. Elsewhere, a leaflet described “a bloodbath in Europe in the name of the freedom and honour of the German nation.” In 1942, the first leaflet said [in what is one of the few, or possibly only, public condemnations of the Holocaust by a German resistance group], “who of us can tell the ignominy that will face us and our children when the veil has fallen from our eyes and the most gruesome and immeasurable crimes come to light?” (The Times, 22 February 2018)

➢ China has the world’s largest population of people with rare diseases, with an estimated 15-20 million sufferers. With limited health care coverage for rare diseases, people face bills that are an average 3 times higher than their household incomes, and parents are spending their life savings or fundraising to pay for their children’s treatments. There is a shortage of orphan drugs [a drug developed specifically to treat a rare medical condition], with only 38% of orphan drugs available in the USA being available in China. Huang Rufan, founder of the Chinese Organisation for Rare Diseases, calls for wider government insurance, stating that it is the most urgent need for patients with rare diseases. However, there are some indications that more support may be forthcoming, as in 2017 the government announced plans to draw up its first initial list of rare diseases which could lead to wider financing of treatment. In addition, some wealthier provinces are beginning to add rare disease drugs to their reimbursements lists, and the China Food and Drug Administration has vowed to speed up the process for approving new treatments. (Financial Times, 28 February 2018)

## Science and Technology

➢ According to a ruling from the European Court of Justice, crops and pharmaceuticals created using gene-editing techniques such as CRISPR-Cas9 may not require regulations within the strict EU rules that govern genetically modified organisms (GMOs). Gene edited allows tiny changes to be made to a genome in a controlled manner to create sturdier crops or improve medical treatments, and scientists have cautiously welcomed the ruling. If gene-editing is deemed to be subject to the same legislation as GMOs, it could be too expensive to develop such products for the European market, and they could potentially be regarded as GMOs by the public, and risk rejection. Globalisation and climate change could bring new diseases to Europe, and gene editing could avert any disastrous consequences, plus allow for crops to be effectively adapted. (Nature, 19 January 2018)

➢ Three of the USA’s giant companies [Amazon, JP Morgan Chase and Berkshire Hathaway] announced plans to shake up American health care by experimenting with their employees’ coverage. They envisage using technology to provide simplified and affordable medical care, eg, creating online services for medical advice or negotiating lower drug prices. However, technology companies in China (eg, Alibaba and Tencent) have long focused on health care, and after testing online medical advice and drug tracking systems, are now targeting artificial intelligence (AI). AI is a potential solution to China’s overburdened hospitals, a lack of primary health care, unequally distributed medical resources, low doctor/patient ratios, a rapidly aging population, high rates of childhood obesity and diabetes, and government plans to extend health care provision. The move towards AI in health care is strongly supported by China’s government, which plans for China to be a leader in this field by 2030. Moreover, China’s data protection and privacy laws are less stringent that those in the USA, allowing for easier data collection that could result in more effective AI systems, coupled with the sheer size of data available from its 1.4 billion people. Venture capitalists are investing heavily in China’s AI health care sector, with companies such as Sequoia and Matrix Partners investing at least US$ 2.7 billion, and it is estimated the spending in China’s health tech industry will reach US$ 150 billion by 2020. (New York Times, 31 January 2018)

➢ Unicode is the governing body for emojis, and new emoji designs are adopted following global crowdsourcing. Emojis are largely used to communicate nouns, as adjectives and – to a lesser extent – verbs – are almost impossible to convey, but the meaning of nouns can be precisely conveyed. Their meaning is universal, and can transcend language and literacy barriers. The latest collection of emojis adopted by Unicode includes a mosquito emoji, which was developed by Jeff Chertack (Bill and Melinda Gates Foundation), and Marla Shaivitz (Johns Hopkins Center for Communication Programs), which can be used for public health messages. It would give health professionals a quick and universal way to communicate with the public about the presence of mosquitoes, and enable researchers to promote their work on mosquito-borne diseases more easily via social media. (Wired, 12 February 2018)

➢ A machine learning system (Artificial Intelligence in Medical Epidemiology, or AIME) may be able to predict dengue outbreaks up to three months in advance, and is being rolled out in a Malaysian state; and several other cities across Asia and Latin America are also trialling the system. The system uses hundreds of parameters, ranging from wind speed to local roof architecture, and then advises on the intervention likely to be most effective in that particular area, such as fogging or removing water pools. As doctors send in notifications of dengue disease, they are logged automatically in AIME, which then searches over 90 databases for 276 variables that influence the spread of dengue. The prevalence of dengue fever, a mosquito-borne viral infection, has grown rapidly in recent decades, with 50% of the world’s population at risk, and in Asia, vector control costs US$ 300 million each year, and US$ 1 billion in Latin America. Dhesi Raja, one of the system’s co-inventors, said that AIME was developed out of frustration at the current “passive, reactive” way of managing vector-borne diseases. “We have good measures like fumigation, larvicides, GM mosquitoes, we even have Wolbachia [bacteria that reduce the ability of insects to become infected with viruses], but the point is if we do not know when and where this outbreak might occur we spend a lot on unplanned management and nationwide campaigns,” he said. (SciDevNet, 2 May 2018)

➢ The USA has enacted new a “Right to Try” law, which will allow terminally-ill patients to try experimental treatments outside of clinical trials without Food or Drug Administration (FDA) approval, as long as drug companies are willing to provide them. The new law weakens the FDA’s power over unproven drugs, and supporters argue that it is necessary as it is currently very cumbersome for patients and doctors to get FDA approval to try new drugs. However, the FDA already approves most of these requests and its supporters say that FDA oversight is needed to protect patients, and that “Right to Try” does not require companies to provide experimental drugs. Companies will probably see the value of FDA affirmative adjudication over patient requests for experimental treatments, and the current “compassionate use program” will likely sit alongside “Right to Try”, as the FDA generally has compelling reasons for the handful of applications it rejects. (Wall Street Journal, 3 June 2018)

